# Injury prevention knowledge, beliefs, and practices among women’s football teams in South Africa

**DOI:** 10.17159/2078-516X/2021/v33i1a9505

**Published:** 2021-07-20

**Authors:** U Bakare, B Olivier, C Brandt, L Godlwana

**Affiliations:** 1 Department of Physiotherapy, School of Therapeutic Sciences, Faculty of Health Sciences, University of the Witwatersrand, Johannesburg, South Africa; 2 Wits Sport & Health (WiSH) Research Group, Faculty of Health Sciences, University of the Witwatersrand, Johannesburg, South Africa

**Keywords:** FIFA 11+, soccer, female, sport, implementation

## Abstract

**Background:**

Numerous factors account for injury prevention or lack thereof in any team setting. With the increasing burden of injuries in women’s football, and limited human resources accessible in sub-Saharan Africa, it is important to investigate the ways in which standardised injury prevention practices can be achieved.

**Objectives:**

The study aimed to evaluate injury prevention knowledge, beliefs, and practices in women’s football teams in the University Sport South Africa (USSA) Football League in Gauteng Province, South Africa.

**Methods:**

A cross-sectional self-administered survey was conducted among women’s football teams registered to participate in the USSA Football League in South Africa’s Gauteng Province.

**Results:**

A total of 107 respondents participated in the study, which included both players (n=98; 92%) and their support staff (n=9; 9%). The median (interquartile range) age of the participants was 22 (20–25) years. In the population sampled, 36% of the participants perceived that they had adequate knowledge of injury prevention practices in football, while others felt they had limited knowledge of the basic injury prevention programmes (IPPs). The results also indicated that the injury prevention practices of coaches (93%) and their beliefs in this regard (70%) are sufficient for achieving the basic injury prevention goals. Most of the respondents (89%) indicated that a medical support system is important in attaining the goals of injury prevention.

**Conclusion:**

Members of women’s teams in the USSA Football League have recognised limited knowledge about the basic IPPs, while they do employ some of the basic injury prevention practices in football. These practices could be influenced by the beliefs of the coaches and the players, and most of them believe that IPPs are important. It is essential as key stakeholders that coaches’ and players’ education and knowledge of injury prevention strategies should be considered as an integral part of the process to succeed. It should be strongly highlighted and implemented, thus augmenting the credibility, trust and compliance for IPPs in the sport.

Football is the world’s most popular sport, with an estimated 300 million registered players globally.^[1]^ Regardless of the level of professionalism, football-related injuries are common. Also, irrespective of gender, the most common injuries reported in football are knee, hamstring, groin and ankle injuries.^[2, 3]^Apart from the common injuries associated with men’s and women’s football, there are also other specific injuries, such as anterior cruciate ligament (ACL) injuries, that are sustained mainly by women.^[2]^ Owing to the increased Q-angle and increased joint laxity arising from their particular hormone status and waist-hip ratio,.^[2]^ women have been found to be at higher risk of presenting with ACL injuries.

The estimate of 30 million women players across the globe is an indication of the significance of their participation in football. As reported by the Fédération Internationale de Football Association (FIFA), about 4.8 million women are registered by national federations worldwide. ^[4]^ The Women’s Football Survey of 2014 shows that the Confederation of African Football (CAF) comprises 1 255 400 members and these numbers have been projected to continue to rise.^[4]^ In an annual European study of the Professional Women’s League in Germany, 246 injuries were reported, thus accounting for an injury rate of 3.3 per 1 000 hours (games: 18.5 per 1 000 hours; practice: 1.4 per 1 000 hours).^[5]^ Injury prevention at any level in football is of utmost importance given the challenges of reduced performance, adverse financial impact, team success and the long-term health of the players.^[6]^ Structured generalised warm-up programmes have been shown to prevent common injuries in football ^[7]^ Although injury prevention programmes (IPPs) are highly effective in structured randomised control trials (RCTs) and prospective controlled studies, this does not guarantee their effectiveness in the real-world context of implementation.^[7]^ In addition to the knowledge of the various IPPs, their permutations and strategies, a better understanding of the implementation context is important for success in this regard in the real world.^[7]^

Injury prevention in sports holistically entails using structured interventions and a multidisciplinary approach to improve the intrinsic and extrinsic risk components to optimise performance and protect the health of players.^[8]^ Some of these intrinsic factors include flexibility, endurance, strength, agility, coordination, and balance, while extrinsic factors include the kits of the players, the available equipment and training surfaces, weather conditions, and the rules of the game. Prevention strategies are comprised of but are not limited to interventions that involve training and prehabilitation. Both include neuromuscular exercises for warm-ups and cool-downs, flexibility, agility, strength, and conditioning. Other considerations include hydration, nutrition, and psychotherapy.^[8, 9]^

Compared to other sports, football is a vigorous sporting activity, with a relatively high incidence of injury, so that preventative programmes to reduce the number of injuries, limit early retirement, and provide a healthy and safe environment for players, are highly recommended.^[10]^ Based on the current literature, the FIFA 11+ comprehensive warm-up programme is a leading IPP which has been proven in scientific studies to significantly mitigate the burden of injuries and reduce time-loss in football.^[11]^ Previous studies were carried out mainly in settings associated with superior human and infrastructural resources, and the results are therefore not comparable to those in sub-Saharan Africa generally.^[12–14]^ The FIFA 11+ programme was first implemented on a large scale in a women’s football population in Norway.^[14]^ Thereafter, the programme was implemented and tested on mainly male football populations in countries such as the Netherlands, ^[15]^ Nigeria, ^[16]^USA ^[13]^ and Australia.^[12]^ There is generally a dearth of knowledge on injury prevention, injury prevention practices and implementation studies on the African continent. In 2014, in their research conducted in Canada, McKay et al. concluded that gaps in injury knowledge and beliefs differ for coaches and players.^[17]^

A paradigm shift in the realm of injury prevention in football and other sports now manifests as a global phenomenon that has made a substantial positive public health impact in that it reduces the burden of injuries, thereby optimising performance and subsequently translates into team success. To understand the existing body of knowledge and practices of injury prevention, it is important to identify the core components if the relevant implementation strategies are to succeed. Each environment and each demographic is unique, considering the existing parameters that could promote compliance in attaining the injury prevention goals, which require access to the current baseline knowledge and the practices that can be accessed.^[18]^ To enhance the ultimate impact of team ball sport exercise-based IPPs, the future reporting of exercise-based IPP trials must clearly address all aspects of programme delivery, as outlined by the Reach Effectiveness Adoption Implementation Maintenance (RE-AIM) framework.^[18]^

Apart from the implementation of a comprehensive IPP, some basic injury prevention principles, such as warm-ups, stretching, cool-downs and jogging, are also beneficial in reducing injuries in football. Women’s football is currently in a developing phase, and there have recently been reports of local professional leagues gathering momentum in South Africa. With limited resources and access to healthcare facilities proving to be expensive, injury prevention strategies will help to lighten some of the burdens associated with participation in football.

The aim of our study was to evaluate the knowledge, beliefs and practices pertaining to injury prevention among members of women’s football teams in the USSA Football League in Gauteng Province, South Africa.

## Methods

### Study design and setting

This study is a cross-sectional survey of injury prevention knowledge and practices among women’s football teams in the USSA Football league in Gauteng Province, South Africa.

### Participants

All women’s teams and their support staff, consisting mainly of head coaches, assistant coaches, team managers and occasional medical staff in the USSA Football League in Gauteng, were invited to participate. The USSA Football League is a competitive football competition consisting of university teams playing in a league format. Ten women’s football teams were eligible to participate in this study, consisting of an estimated population of 180 players and 20 coaches and support staff. All participants who gave their written informed consent were eligible to participate.

### Ethical consideration

The University of the Witwatersrand Human Research Ethics Committee (Medical) approved the study (M161146). Permission was granted by the USSA Football League and all the participating football team managers. All participants read, understood, and signed a written informed consent form.

### Questionnaire

The self-administered questionnaire was adapted from a study by McKay et al. ^[17]^ The questions included the domains of socio-demographic attributes, playing and training characteristics, injury prevention practices, other injury prevention aspects and associated beliefs. The questionnaire was structured to include a dichotomous ‘Yes’ and ‘No’ two-point scale, while a five-point Likert scale was used to measure agreement across the extremes of ‘strongly agree’ to ‘strongly disagree’. The questionnaire was validated for content and face validity with sports medicine experts and was piloted with an amateur male football team that did not form part of the study.

### Procedure

A total of 10 teams regularly participates in the USSA Football League, and of the ten teams, seven teams consented to participate in the study. Informed consent was sought from the individual players and support staff, and the questionnaire was administered at the respective practice venues for each of the teams. Data were collected through site visits to training sessions between July 2017 and March 2018. Appointments were scheduled with the respective team managements for the researcher to administer the questionnaires.

### Statistical analysis

Data analysis was performed using the IBM SPSS 25.0® programme. The Shapiro-Wilk test was used to determine the normal distribution of the continuous data i.e. age and years of experience. Descriptive statistics of the median and interquartile range (IQR) were used to summarise the continuous data, as these data were not normally distributed. The interquartile range was presented as the difference between the 75^th^ and 25^th^ percentiles.^[19]^ Percentages and frequencies were used to summarise the categorical variables. Missing data were regarded as missing.

## Results

The estimated population size was initially envisaged to be 200 participants, but only 54% (n=107) eventually participated in the study. The median (IQR) age and years of experience of all the participants was found to be 22 (20–25) years and 36 (12–48) months, respectively. The median (IQR) ages of the players and support staff were 22 (20–24) years and 32 (25–39) years, respectively. The socio-demographic characteristics of the participants are outlined in Table 1.

Of the 107 participants, 36% (n=38) reported they perceived that they had an adequate knowledge of injury prevention. It was also shown that 76% (n=81) of the participants were engaged in football IPP at the time of the research, with the following injury prevention practices being relevant: warm-ups (95%), stretching (90%), cool-downs (80%) and jogging (81%). Most of the participants (67%) generally engage in injury prevention practices daily and 87% reported carrying out injury prevention practices during matches and team training sessions (70%). Only 25% of the respondents indicated that they had heard about the FIFA 11+ IPP, while the majority (83%) stated that they would be willing to adopt it if they were to gain access to detailed information on the programme (Table 2).

In Table 3, most participants (87%) strongly agreed that IPPs are important, and 66% believed that medical support is important in achieving injury prevention goals. Most of the participants (65%) also strongly agreed that their coach’s prevention practices are sufficient for attaining injury prevention goals. A considerable number of the participants reported ‘Don’t know’ in the following instances: African beliefs significantly affect injury prevention practices (41%), and the coach’s beliefs are important in achieving injury prevention goals (18%). It was also shown that the majority (66%) of the respondents indicated that a medical support system is important in achieving the injury prevention goals (Table 3).

## Discussion

### Knowledge of injury prevention practices

In our study, only 36% of the participants reported that they perceived themselves as having adequate knowledge of basic injury prevention practices in football. Other studies on injury prevention have reported similar results.^[17, 20]^ In the research conducted in Canada by McKay et al.^[17]^ they showed a dearth in the perception of knowledge and beliefs in young amateur female soccer players, emphasising effective prevention strategies and injury risk factors, but with significant differences in feedback from coaches and players. A study conducted in Nigeria among a youth football league reported that most players fall within the poor and fair knowledge categories.^[20]^ Another study in South Africa, which evaluated the coach’s knowledge, attitude, and perceptions regarding injury prevention among amateur footballers, showed that knowledge perception about injury prevention in coaches is limited.^[21]^ This indicates that knowledge may still be a challenge irrespective of geographical location. However, in a study by Geertsema et al.^[22]^ conducted to evaluate knowledge, beliefs and strategies in a cohort of players at the FIFA Women’s World Cup, concluded that elite female footballers reported awareness of the level of risk and the types of injuries in women’s football. The authors also suggested that players hold positive attitudes towards injury prevention. The good knowledge score demonstrated in the study by Geertsema et al.^[22]^ might be because the study was conducted amongst elite female footballers who have access to better economic resources and support staff.

The perceptions of knowledge concerning football injury prevention reported in our study are in tandem with those recorded in some previous studies.^[20, 23]^ In the South African context, this might be because the participants are primarily students and amateur players. In addition, there is limited infrastructural and financial support in the growing women’s football league in South Africa.^[24]^ A systematic review conducted by Al Attar et al.^[11]^ concluded that adequate knowledge about injury prevention in football reduces the incidence of injury and improves performance. Studies that have implemented exercise-based IPPs in sports have recorded a significant reduction in injury incidence.^[25]^ Therefore, it is imperative that adopting and implementing a standard IPP needs to take into account and include the improvement of the knowledge base of the programme by educating the players and coaches about injury prevention practices and injury risk factors. Thus, adequate education in injury prevention strategies directed at all role players, particularly the coaches, is paramount.^[25]^

### Injury prevention practices employed by members of women’s football teams

The common injury prevention practices reported by participants include warm-ups, cool-downs, jogging, and stretching. These injury prevention practices have also been reported in the literature to reduce the incidence of football-related injuries.^[2, 11]^ However, a more comprehensive warm-up protocol that encompasses all these components has been developed and its efficacy has been proven in several studies.^[11]^ Warms-ups physically prepare athletes for the competition, and influence their mental state of mind, which is crucial to optimising performance. A well-designed warm-up programme will achieve physiological responses in the cardiovascular, musculoskeletal, respiratory, nervous, and metabolic systems.^[26]^ These responses include a raised tissue temperature, increased circulation to working muscles, and enhanced oxygen supply from the oxy-haemoglobin and myoglobin breakdown. Cool-downs are the first step in the recovery process, the gradual tapering down of activities from an intense session of exercise, and the commencement of a ‘reverse phase’ of the warm-up process. A cool-down is effectively the tapering-off phase at the end of explosive physical activity and is typically at low intensity.^[26]^

Another key component is stretching, which may be static or dynamic. Stretching aims to return muscles that have typically been shortened because of contractions during explosive sporting activity back to their resting length or their pre-activity state.^[26]^ Although it is important to note that these injury prevention practices are helpful in isolation, each component combined with the others will constitute a concerted effort towards achieving the injury prevention goals. Very few of the participants in our study were aware of the FIFA 11+, which is a comprehensive injury prevention programme, but the majority were keen to learn more about it and adopt it.

### Beliefs about injury prevention

From our results, coaches and players believe IPPs work, and that they would be willing to adopt them. It has been shown that the coach plays the most important role in adopting and implementing injury prevention practices.^[25]^ The majority of the participants identified that their coach’s beliefs are important in achieving injury prevention goals, and they believe that compliance is a key component of IPPs. Interestingly, in our study, the participants expressed that their coach’s IPP strategy is adequate even though only 36% reported that their particular knowledge about injury prevention in football is based on their own perceptions. This is not surprising, as often in sports, the relationship between the coach and the player follows a one-directional pattern where the coach is considered as the mentor, leader and guide in the career of the players.^[25]^ In addition, the majority of the coaches are males, and the female-male dynamic element in sports usually influences the communication strategy and interpretation.^[27]^ When a coach is keen on a programme, he/she will tend to influence the team’s knowledge and training regarding injury prevention, hence achieving the injury prevention goals.

Professional women’s football in South Africa is in the developmental stage, with the amateur leagues showing considerable improvement in terms of personnel and infrastructure.^[24]^ Currently, because of limited personnel, the coach plays multiple roles in managing the team by providing medical support, leading the strength and conditioning programmes, and the injury prevention practices. This study also shows the lack of adequate medical personnel available on the team’s support staff, which might be largely due to the economic circumstances of the team. Therefore, it is pertinent that for the successful implementation of an IPP strategy in a low- to middle-income setting in countries such as South Africa, the coach will play a pivotal and integral role in the implementation plan, adoption, compliance, and overall implementation of such a programme.

### Limitations

This study was limited to women’s football teams in the USSA Football League in Gauteng Province; therefore, the findings should not be generalised to other populations, sports, and demographics. The questionnaire used in this study was self-administered and is based on the individual perceptions of participants and therefore might be subject to bias.

### Recommendations

The potential burden of injuries will continue to grow within the current environment and the increasing participatory numbers in women’s football in South Africa. To mitigate this burden of injuries associated with football participation, it would be pertinent to incorporate evidence-based IPPs to achieve the desired goals of injury prevention. There needs to be a paradigm shift to the effect that the adoption and implementation of holistic injury prevention practices would reduce the incidence of injury and improve the performance of the players and the overall achievements of their teams. As key stakeholders, coaches’ and players’ education and knowledge of injury prevention strategies should be considered an integral part of the process to succeed, strongly highlighted and implemented, thus augmenting the credibility, trust, and compliance for IPPs in the sport. Proper understanding might lead to a simple screening process that would be meaningful for IPPs. It is also important to ensure that medical support should be an integral part of the process of achieving the injury prevention goals. Therefore, as recommended by the RE-AIM framework for implementation, it is important to be purposeful and devise an implementation strategy for IPPs.

## Conclusion

Most members of women’s football teams perceived their level of knowledge as inadequate. Although the respondents engage in various injury prevention practices, such as warm-ups, stretching, cool-downs and jogging, very few are aware of the FIFA 11+ Football IPP. Most players and coaches believe that IPPs are essential, which are crucial first steps towards developing and implementing injury prevention awareness programmes.

## Figures and Tables

**Table 1:** Socio-demographic characteristics of the participants (n=107)

Characteristics	n (%)

**Gender**	
Female	102 (95)
Male	5 (5)
**Designation**	
Assistant coach	4 (4)
Head coach	3 (3)
Player	98 (92)
Team Manager	2 (2)
**Team**	
North West-Vaal	15 (14)
Sefako Makgatho University	9 (8)
Tswane University of Technology	8 (8)
University of Johannesburg	22 (21)
University of Pretoria	24 (22)
Vaal University of Technology	9 (8)
Wits University	20 (19)
**Player position (n=98)** *	
Defender	41 (42)
Goalkeeper	11 (11)
Midfielder	21 (21)
Striker	25 (26)

* Player position is number of players, less number of support staff.

**Table 2:** Injury prevention knowledge and practices (n=107)

Questions	n (%)

Do you engage in any football injury prevention practices? (Yes)	81 (76)
Type of injury prevent practices*	
Warm-ups	77 (95)
Stretching	73 (90)
Cool-downs	65 (80)
Jogging	66 (81)
Did you receive any training in the injury prevention practices that you are currently engaged in? * (Yes)	59 (72)
How many times do you engage in the injury prevention practices that you use? *	
Daily	54 (67)
Weekly	17 (21)
Monthly	10 (12)
Frequency per week∞	
Once	24 (30)
Twice	26 (32)
Thrice	11 (14)
Other	18 (22)
When do you carry out IPPs? *	
Match days	7 (9)
Training	17 (21)
Both	57 (70)
During which training session do you carry out IPP? *	
Self-training	11 (14)
Team training	60 (74)
Both	10 (12)
Are your injury prevention choices based on your personal beliefs about preventing injury? † (Yes)	35(33)
Do you think that the injury prevention practices that you engage in are based on scientific research? † (Yes)	56 (52)
Do you think that you would be willing to adopt a different injury prevention strategy from that which you are already engaged in? † (Yes)	64 (60)
Do you think that your knowledge about injury prevention in soccer is adequate? † (Yes)	38 (36)
Do you think that you are effectively applying this knowledge as a football player/coach? † (Yes)	65 (61
Do you think that your current injury prevention strategy works? † (Yes)	52 (49)
Have you heard about the FIFA 11+ Football IPP? † (Yes)	26 (25)
Are you interested in adopting the FIFA 11+ Football IPP? † (Yes)	89 (83)

* ; n=81, percentage based on participants that answered ‘Yes’ to the preceding question “Do you engage in any football injury prevention?”.

∞ ; n=79, missing data, less participants who did not complete the question in the self-administered questionnaire.

† ; n=107, percentage based on the total number of participants.

**Table 3:** Injury prevention beliefs (n=107)

Beliefs		Strongly agree	Agree	Don’t know	Disagree	Strongly disagree
1	Injury prevention practices are important in football.	93(87)	9(8)	1(1)	2(2)	2(2)
2	A medical support system is important in achieving the goals of injury prevention.	71(66)	24(22)	7(7)	3(3)	2(2)
3	The coach’s injury prevention practices are sufficient for achieving injury prevention goals.	70(65)	29(27)	6(6)	-	2(2)
4	The player’s knowledge is important in achieving injury prevention goals.	67(63)	31(29)	5(5)	2(2)	2(2)
5	Identifying injury risk factors and modifying them contributes to achieving injury prevention goals.	62(58)	33(31)	11(10)	-	1(1)
6	It is key to work as a team to achieve the goals of injury prevention.*	67(63)	36(34)	2(2)	-	1(1)
7	Injury prevention is directly related to team success.	59(55)	35(33)	11(10)	1(1)	1(1)
8	Compliance to an IPP is important in achieving its set goals.	59(55)	40(37)	6(6)	1(1)	1(1)
9	The coach’s beliefs are important in achieving injury prevention goals.*	37(35)	37(35)	19(18)	8(8)	5(5)
10	The FIFA 11+ Football IPP is important in achieving injury prevention goals.*	73(69)	31(29)	2(2)	-	-
11	An implementation guide is important in achieving injury prevention goals.*	56(53)	38(36)	11(11)	-	-
12	African beliefs significantly affect injury prevention practices.*	26(25)	20(19)	44(42)	9(9)	6(6)
13	Injury prevention practices enhance performance optimisation.*	56(54)	31(30)	17(16)	-	-

Data displayed as n (%).

* indicates missing data (Q6, Q7 and Q 10: n=106); (Q11 and Q12: n=105); Q13: n=104) – less participants who did not complete the question in the self-administered questionnaire.
